# Chromosomal localization of Ewing sarcoma EWSR1/FLI1 protein promotes the induction of aneuploidy

**DOI:** 10.1074/jbc.RA120.014328

**Published:** 2020-12-10

**Authors:** Hyewon Park, Haeyoung Kim, Victoria Hassebroek, Yoshiaki Azuma, Chad Slawson, Mizuki Azuma

**Affiliations:** 1 Department of Molecular Biosciences, University of Kansas, Lawrence, Kansas, USA; 2 Department of Biochemistry and Molecular Biology, University of Kansas Medical Center, Kansas City Kansas, USA

**Keywords:** Ewing sarcoma, EWSR1/FLI1, Aurora B, aneuploidy, mitosis, CIN, chromosome instability, Dox, doxycycline, hMSC, human mesenchymal stem cells, HSD, honestly significant difference, PTM, posttranslational modification, SAC, spindle assembly checkpoint

## Abstract

Ewing sarcoma is a pediatric bone cancer that expresses the chimeric protein EWSR1/FLI1. We previously demonstrated that EWSR1/FLI1 impairs the localization of Aurora B kinase to the midzone (the midline structure located between segregating chromosomes) during anaphase. While localization of Aurora B is essential for faithful cell division, it is unknown whether interference with midzone organization by EWSR1/FLI1 induces aneuploidy. To address this, we generated stable Tet-on inducible cell lines with *EWSR1/FLI1,* using CRISPR/Cas9 technology to integrate the transgene at the safe-harbor AAVS1 locus in DLD-1 cells. Induced cells expressing EWSR1/FLI1 displayed an increased incidence of aberrant localization of Aurora B, and greater levels of aneuploidy, compared with noninduced cells. Furthermore, the expression of EWSR1/FLI1-T79A, containing a threonine (Thr) to alanine (Ala) substitution at amino acid 79, failed to induce these phenotypes, indicating that Thr 79 is critical for EWSR1/FLI1 interference with mitosis. In contrast, the phosphomimetic mutant EWSR1/FLI1-T79D (Thr to aspartic acid (Asp)) retained the high activity as wild-type EWSR1/FLI1. Together, these findings suggest that phosphorylation of EWSR1/FLI1 at Thr 79 promotes the colocalization of EWSR1/FLI1 and Aurora B on the chromosomes during prophase and metaphase and, in addition, impairs the localization of Aurora B during anaphase, leading to induction of aneuploidy. This is the first demonstration of the mechanism for EWSR1/FLI1-dependent induction of aneuploidy associated with mitotic dysfunction and the identification of the phosphorylation of the Thr 79 of EWSR1/FLI1 as a critical residue required for this induction.

Ewing sarcoma is a pediatric bone cancer that displays a characteristic small round blue cell morphology (1). An early investigation of the disease demonstrated that patient cells express an aberrant *EWSR1/FLI1* fusion gene composed of the N-terminal coding region of the EWS RNA Binding Protein 1/Ewing Sarcoma Breakpoint Region 1 (*EWSR1*) gene and the C-terminal coding region of the FLI-1 Proto-Oncogene, ETS Transcription Factor (*FLI1*) gene (2, 3). Subsequent studies demonstrated that 85% of Ewing sarcoma patients have tumors that express the *EWSR1/FLI1* fusion gene, while the remaining patients have tumors that express fusion genes composed of *EWSR1* and other ETS transcription factors (*ETV1, ETV4, ERG,* and *FEV1*) (4, 5, 6, 7, 8). In all cases, the *EWSR1* fusion genes encode the transactivation domain from EWSR1 and the ETS DNA binding domain from the ETS transcription factor. The reported effects of EWSR1 fusion protein expression include: 1) altered epigenetic status due to aberrant recruitment of the chromatin remodeling complex, and 2) aberrant expression of target genes in Ewing sarcoma cells (9, 10, 11, 12, 13, 14, 15). In addition, several reports describe misregulated gene expression resulting from impaired splicing due to EWSR1/FLI1 (16, 17, 18).

Ewing sarcoma is known to carry quiet genome, a relatively low number of secondary DNA mutations (19, 20, 21). The genomics of Ewing sarcomas have been extensively studied, and several genes have been identified as frequently mutated in these tumors. For example, mutations in *STAG2* have been identified in 17% of patients, mutations in *CDKN2A* were found in 12% of patients, and mutations in *TP53* were found in 7% of patients (19, 20). While the mutations in these genes are thought to play an important role in disease progression, the low incidence of secondary mutations suggests the existence of an alternative mechanism driving pathogenesis. One understudied phenomenon is the high incidence of aneuploidy observed in Ewing sarcoma. These changes include the gain of chromosome 8 in 47% of patients, the gain of chromosome 21 in 21% of patients, and the loss of part of chromosome 16 in 17% of patients (20, 22). Although the tumor cells display a high incidence of aneuploidy, it is unclear whether aneuploidy is induced in the early stages of tumorigenesis and promotes the progression of the disease, or if it is a by-product of tumorigenesis and expands during tumor progression. Aneuploidy is the result of abnormal chromosome segregation, which may be caused by the failure of any of several mitotic processes (including chromosome cohesion; mitotic spindle (K-fiber) attachment to the kinetochore of the chromosome; the spindle assembly checkpoint (SAC); the amplification of centrosomes; or cytokinesis accompanied by defects in midbody structure) (23, 24). We previously demonstrated that EWSR1/FLI1 impairs the recruitment of the key mitotic regulator Aurora B kinase to the midzone, (the network of antiparallel microtubules and midzone proteins located between segregating chromosomes during anaphase) (25). It is known that impaired localization of Aurora B to the midzone leads to multiple defects, including flawed cytokinesis due to structural defects in the midbody, uneven chromosome segregation, and the induction of aneuploidy (26, 27, 28). Therefore, mitotic dysfunction induced by EWSR1/FLI1 may play a critical role in the induction of Ewing sarcoma.

To begin our investigations into the induction of aneuploidy by EWSR1/FLI1, we generated Tet-on EWSR1/FLI1 inducible cell lines using the CRISPR/Cas 9 system (29). These cell lines allowed us to coordinate the phase of the cell cycle and the expression of EWSR1/FLI1. Here, we demonstrate that EWSR1/FLI1 expression leads to impaired Aurora B-localization and to the induction of aneuploidy after 1 cell cycle after induction of EWSR1/FLI1 expression, suggesting that it is an early event in disease pathogenesis. Furthermore, amino acid residue Thr 79 (T79) of EWSR1/FLI1 is essential for 1) colocalization of the fusion protein with Aurora B during metaphase; 2) impaired localization of Aurora B during anaphase; and 3) the induction of aneuploidy. Surprisingly, substitution of amino acid T79 of EWSR1/FLI1 with alanine abolished the ability of the cells to induce these phenotypes, whereas expression of EWSR1/FLI1-T79D (containing the phosphomimetic substitution of Asp for Thr 79) retained the high activity as wild-type EWSR1/FLI1. These results suggest that phosphorylation of Thr 79 of EWSR1/FLI1 is critical for induction of aneuploidy, likely by facilitating the localization of the fusion protein on the chromosomes, leading to mitotic dysfunction. Our discovery may provide a mechanism to account for the high incidence of aneuploidy observed in Ewing sarcoma and thus contributes to the understanding of the pathogenesis of this devastating disease.

## Results

### EWSR1/FLI1 localizes to the chromosomes at prophase, and residue Thr 79 is critical for this localization

**Figure 1:**
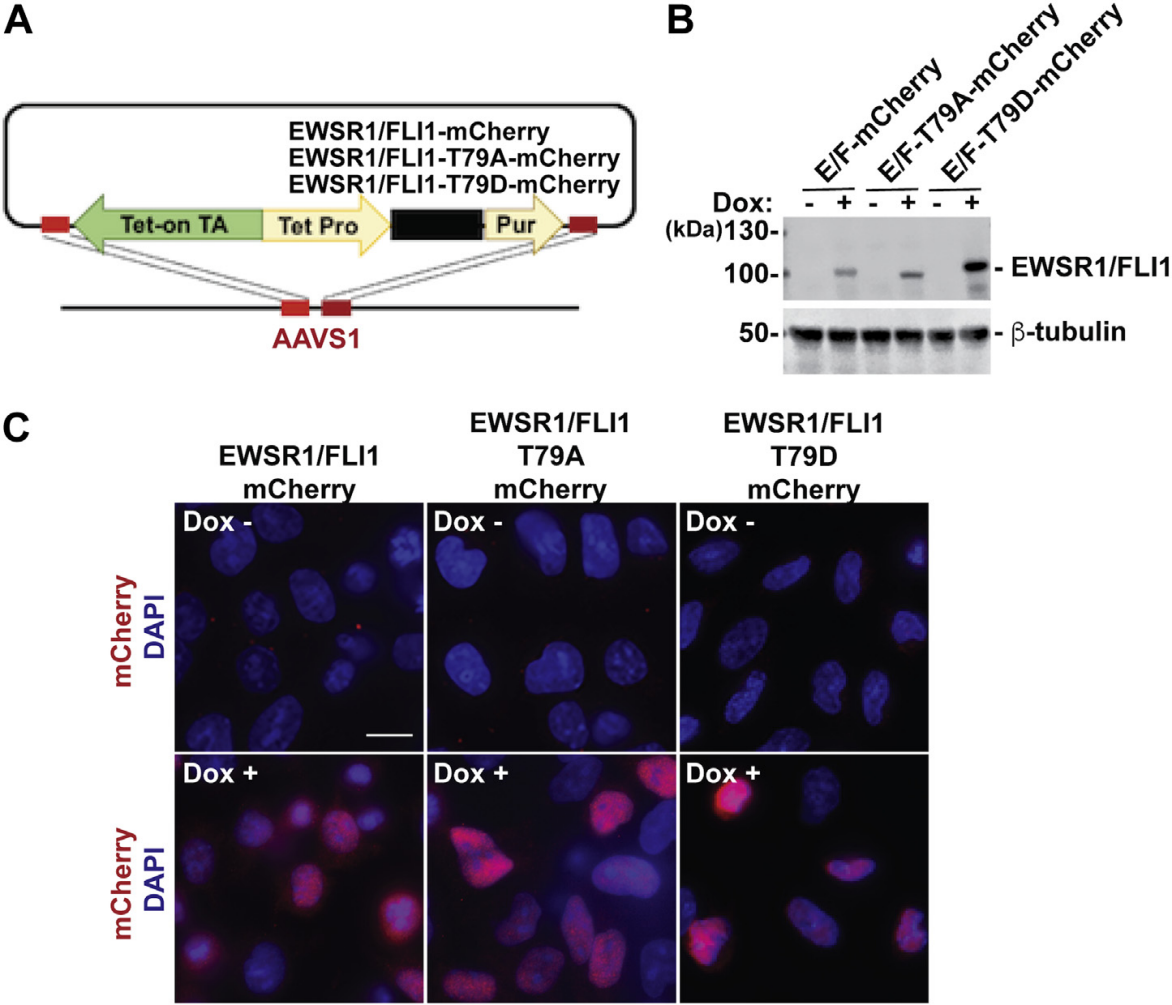
**Establishment of stable DLD-1 cell lines with inducible *EWSR1/FLI1*, *EWSR1/FLI1-T79A*, and *EWSR1/FLI1-T79D***. *A*, Schematic of *EWSR1/FLI1, EWSR1/FLI1-T79A*, and *EWSR1/FLI1-T79D* constructs. Tet-on TA: Tet-on Transactivation, Tet Pro: Tet-on promoter, Pur: Puromycin resistance gene. *B*, The expression of *EWSR1/FLI1-mCherry*, *EWSR1/FLI1-T79A-mCherry*, and *EWSR1/FLI1-T79D-mCherry* in Dox-treated (Dox+) cells and untreated (Dox-) cells was verified by western blot using anti-FLI1 and anti-β-tubulin antibodies. E/F: EWSR1/FLI1. *C*, Representative images of EWSR1/FLI1-mCherry, EWSR1/FLI1-T79A-mCherry, and EWSR1/FLI1-T79D-mCherry expression (*red*) with DAPI (*blue*) obtained from untreated (Dox-) and treated (Dox+) cells. Scale bar = 10um.

To provide insight into the mechanism of EWSR1/FLI1-mediated mitotic dysfunction through impaired Aurora B localization, we initiated our study by identifying the point mutant of EWSR1/FLI1 that lacks the ability to induce aberrant localization of Aurora B. Using mutant constructs to identify the amino acid residues of the fusion protein that are responsible for induction of midzone defects is an effective approach for understanding protein function. Once identified, the critical mutant construct serves as a negative control or positive control in experiments to dissect the pathways involved. Because our previous study showed that the N terminus of EWSR1/FLI1 is required to induce mitotic defects, we used a candidate approach to investigate the critical residues of the fusion protein (30). We investigated residue Thr 79 of EWSR1/FLI1, which is located in the N terminus of the molecule (derived from EWSR1 sequence), because it is a known site for posttranslational modifications. Thr 79 is phosphorylated when cells expressing EWSR1/FLI1 are treated with a DNA-damaging agent (31). Thr 79 is also a potential site for O-GlcNAcylation, which affects the expression of Inhibitor of DNA Binding 2 (ID2) (32). We generated DNA constructs with *EWSR1/FLI1* and with amino acid substitutions at residue 79 in *EWSR1/FLI1.* The substitution constructs had a Thr to Ala substitution (T79A) or a Thr to Asp substitution (T79D; phosphomimetic mutation). Using these three DNA constructs, we established stable Tet-on cell lines by integrating *EWSR1/FLI1*, *EWSR1/FLI1-T79A*, and *EWSR1/FLI1-T79D* transgenes (each with an mCherry tag) into the safe-harbor AAVS1 locus of DLD-1 cells using CRISPR/Cas9 technology (Fig. 1*A*) (29, 33). The DLD-1 colorectal cancer cell line was chosen because it does not express EWSR1/FLI1 endogenously, and because it has a near-diploid karyotype, it has been used as a model to evaluate proteins of interest for their ability to induce aneuploidy (24, 34). The inducible system enabled us to assess the activity of EWSR1/FLI1 in the induction of aneuploidy after a single cell cycle. Expression of EWSR1/FLI1, EWSR1/FLI1-T79A, and EWSR1/FLI1-T79D was verified in induced cells by western blot using an anti-FLI1 antibody and by immunocytochemistry using antibodies against the mCherry tags (Fig. 1, *B–C*).

**Figure 2:**
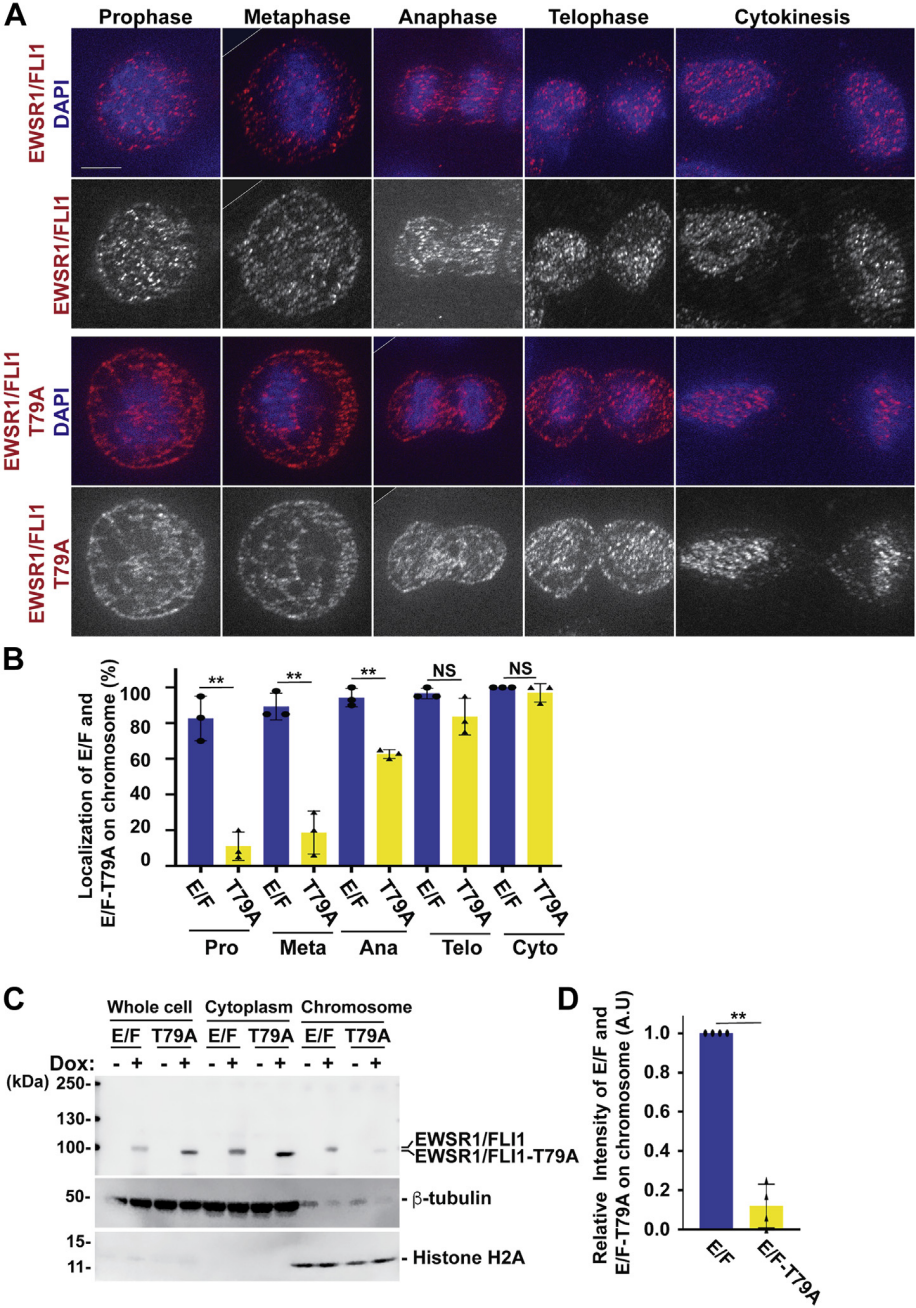
**Localization of EWSR1/FLI1 on chromosomes during early mitosis requires Thr 79**. DLD-1 cells with inducible *EWSR1/FLI1* and *EWSR1/FLI1-T79A* were induced with Dox and synchronized in mitosis using thymidine (*A* and *B*, Fig. S1*A*) or thymidine/nocodazole (*C* and *D*, Fig. S1*B*). *A*, Cells were subjected to immunocytochemistry using anti-mCherry primary and Alexa Fluor 594 conjugated secondary antibodies (*red*), and DNA was stained with DAPI (*blue*). Single Z-sections of cells were captured and representative merged images of mCherry and DAPI signals (top; *red* and *blue*) and mCherry signal alone (bottom; *black* and *white*) show the localization of EWSR1/FLI1 foci on the chromosomes, compared with EWSR1/FLI1-T79A localization in the cytoplasm. The backgrounds of the images were adjusted using the “Brightness” and “Linear-Curves” functions of Photoshop. The edge of rotated image is indicated by thin line. Scale bar = 10um. *B*, Single Z-section images of EWSR1/FLI1 or EWSR1/FLI1-T79A expressing cells were photodocumented, and the percentages of the cells that displayed the localization of either EWSR1/FLI1 or EWSR1/FLI1-T79A on the chromosome was scored. E/F: EWSR1/FLI1, T79A: EWSR1/FLI1-T79A, Pro: Prophase, Meta: Metaphase, Ana: Anaphase, Telo: Telophase, and Cyto: Cytokinesis. *C*, Lysates from whole cells and cytoplasm and chromosome fractions were extracted and subjected to western blot using anti-FLI1 (top panel), anti-β-tubulin (middle panel), and anti-H2A antibodies (bottom). The image was cropped, the color was inverted using the “Invert” function of Photoshop software, and the background was adjusted using the “Brightness” and “Linear-Curves” functions of Photoshop. *D*, Relative intensity of the EWSR1/FLI1 and EWSR1/FLI1-T79A (normalized to Histone H2A) bands from the chromosome sample. (n = 3 experiments). ∗∗*p* < 0.01 (Student’s *t*-test).

We initiated the study by characterizing the EWSR1/FLI1 and EWSR1/FLI1-T79A lines. The cells at mitosis were obtained by using the thymidine and by releasing for 9 h (Fig. S1*A*). First, the localizations of EWSR1/FLI1 and EWSR1/FLI1-T79A were documented using single Z-section images for each sample. In the EWSR1/FLI1 expressing (Dox+) cells, most mCherry positive cells displayed mCherry signal in prominent foci over the chromosomes during prophase and metaphase and signal in the cytoplasm. However, in cells induced to express EWSR1/FLI1-T79A (Dox+), the fluorescent signal in most of the mCherry positive cells was observed less on the chromosomes but was observed mainly in the cytoplasm during both prophase and metaphase (Fig. 2, *A–B*). During anaphase, telophase, and cytokinesis, both the EWSR1/FLI1-mCherry and the EWSR1/FLI1-T79A-mCherry signals localized on the chromosomes (Fig. 2, *A–B*). Together, these results suggest that residue T79 of EWSR1/FLI1 is critical for localization to the chromosomes during early mitosis.

To verify this observation, the localization of EWSR1/FLI1 and EWSR1/FLI1-T79A was further analyzed using a biochemical approach. Both cell lines were treated with Dox (or left untreated), synchronized using the thymidine/nocodazole protocol, and released for 30 min (Fig. S1*B*). The cells were lysed and directly used as whole-cell lysate or fractionated into cytoplasm and chromosome fractions, and all of the samples were subjected to western blot using anti-FLI1 antibody. (Note that DLD-1 cells do not express detectable levels of endogenous FLI1, thus the antibody specifically recognizes both EWSR1/FLI1 and EWSR1/FLI1-T79A). Consistent with the observations using immunocytochemistry, the FLI1 signal appears in the chromosome fraction in Dox treated cells expressing EWSR1/FLI1, and there is virtually no signal in this fraction for the EWSR1/FLI1-T79A (Dox+) sample (Fig. 2*C*). Quantification of the band intensity for the EWSR1/FLI1 and EWSR1/FLI1-T79A signals in the chromosome fractions obtained from three experiments revealed that the intensity of the EWSR1/FLI1-T79A signal was significantly lower than that of EWSR1/FLI1 (defined as 1.0) (Fig. 2*D*). Note that the expression level of the EWSR1/FLI1-T79A is higher than that of EWSR1/FLI1 (Fig. 2*C* whole-cell lysate); however, the localization of EWSR1/FLI1-T79A on the chromosome was significantly lower than that of EWSR1/FLI1. Therefore, the result suggests that the localization of the EWSR1/FLI1 on the chromosome is not regulated by the level of the protein, but the residue Thr 79 is required for EWSR1/FLI1 to localize to chromosomes during mitosis. This is the first evidence that EWSR1/FLI1 is recruited to the chromosome in a Thr 79 site-specific manner.

**Figure 3:**
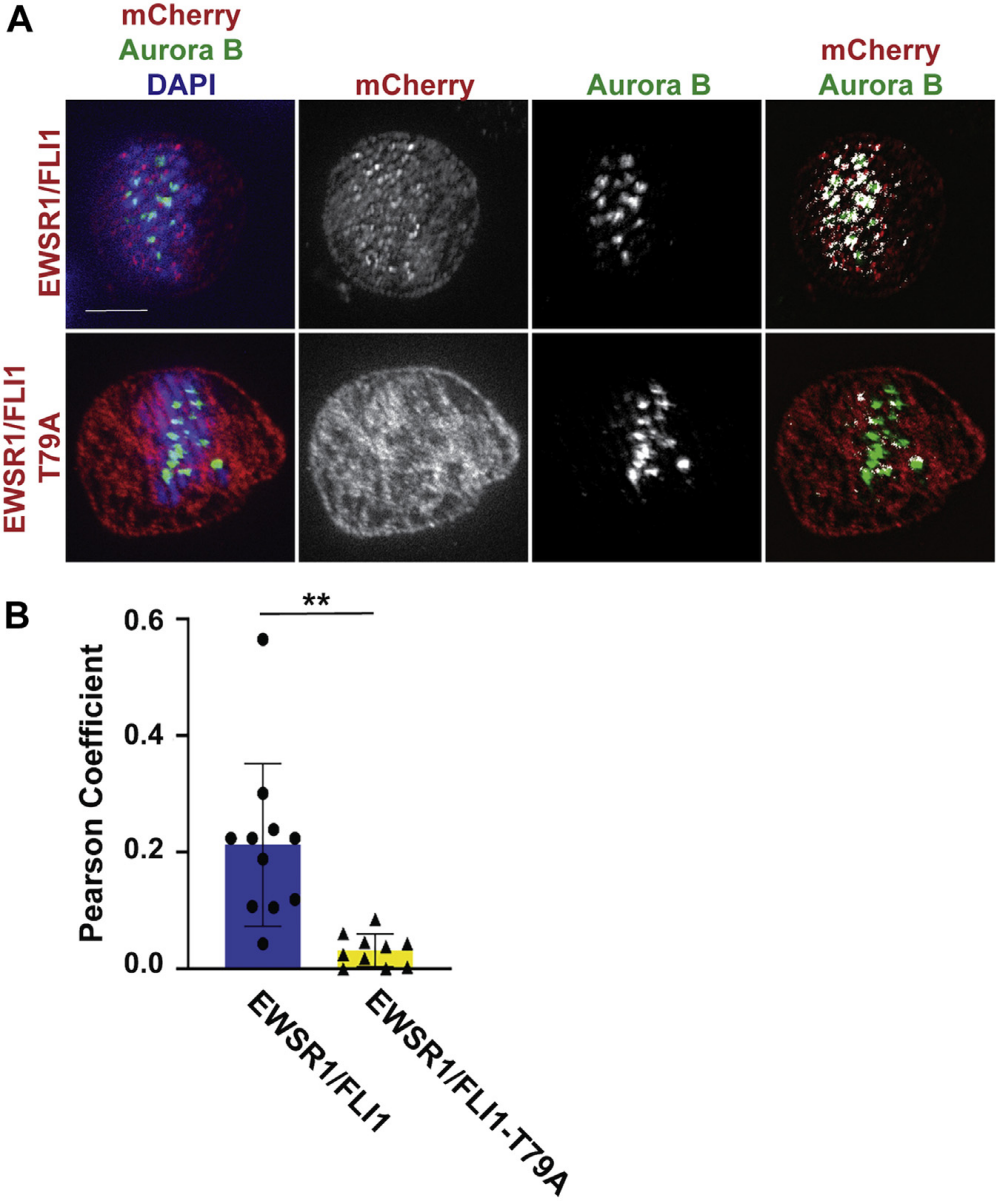
**EWSR1/FLI1-T79 is required for colocalization of EWSR1/FLI1 with Aurora B during metaphase**. The *EWSR1/FLI1* and *EWSR1/FLI1-T79A* cell lines were synchronized in mitosis using thymidine (Fig. S1*A*) and treated with (Dox+), followed by immunocytochemistry using anti-mCherry primary and Alexa Fluor 594 conjugated secondary (*red*), and anti-Aurora B primary and Alexa Fluor 488 conjugated secondary (*green*) antibodies. *A*, Representative images of single Z-sections of cells stained with mCherry/Aurora B/DAPI (left), mCherry (middle), Aurora B (middle), and merged images from mCherry/Aurora B (White represents the areas with colocalized Aurora B (*green*) and mCherry (*red*) signals) (right panel). Scale bar = 10um. *B*, The Pearson Coefficient of Covariance for Aurora B (*green*) and mCherry (*red*) signals in Dox-treated metaphase cells is significantly higher for cells expressing EWSR1/FLI1 (n = 11 cells) than for cells expressing EWSR1/FLI1-T79A (n = 12 cells). ∗∗*p* < 0.01 (Student’s *t*-test).

To address whether the localization of EWSR1/FLI1 on the chromosome correlates with the localization of Aurora B, Dox-treated EWSR1/FLI1 and EWSR1/FLI1-T79A DLD-1 cell lines were prepared by the same thymidine protocol described in Fig. S1*A*. Then, the cells were subjected to immunocytochemistry using anti-mCherry and Alexa Fluor 594 antibodies and anti-Aurora B and Alexa Fluor 488 antibodies. Single Z-section images of the cells were obtained, and areas with overlapping signals for mCherry and Aurora B were analyzed using the pixelmap function of Fiji Imaging software. We identified a significantly broader area of overlapping signals (shown in white in Fig. 3*A*, top row, far-right panel) in EWSR1/FLI1 expressing cells compared with cells expressing EWSR1/FLI1-T79A (Fig. 3*A*, bottom row, far-right panel). The covariance for Aurora B (green) and mCherry (red) signals was determined using the Pearson Coefficient. Dox-treated cells expressing EWSR1/FLI1 (n = 11 cells) show a significantly higher correlation of localization than cells expressing EWSR1/FLI1-T79A (n = 12 cells) (Fig. 3*B*). The result suggests that residue Thr 79 is essential for the colocalization between EWSR1/FLI1 and Aurora B.

### Thr 79 of EWSR1/FLI1 is the critical amino acid required for induction of aberrant localization of Aurora B at the midzone during anaphase

**Figure 4:**
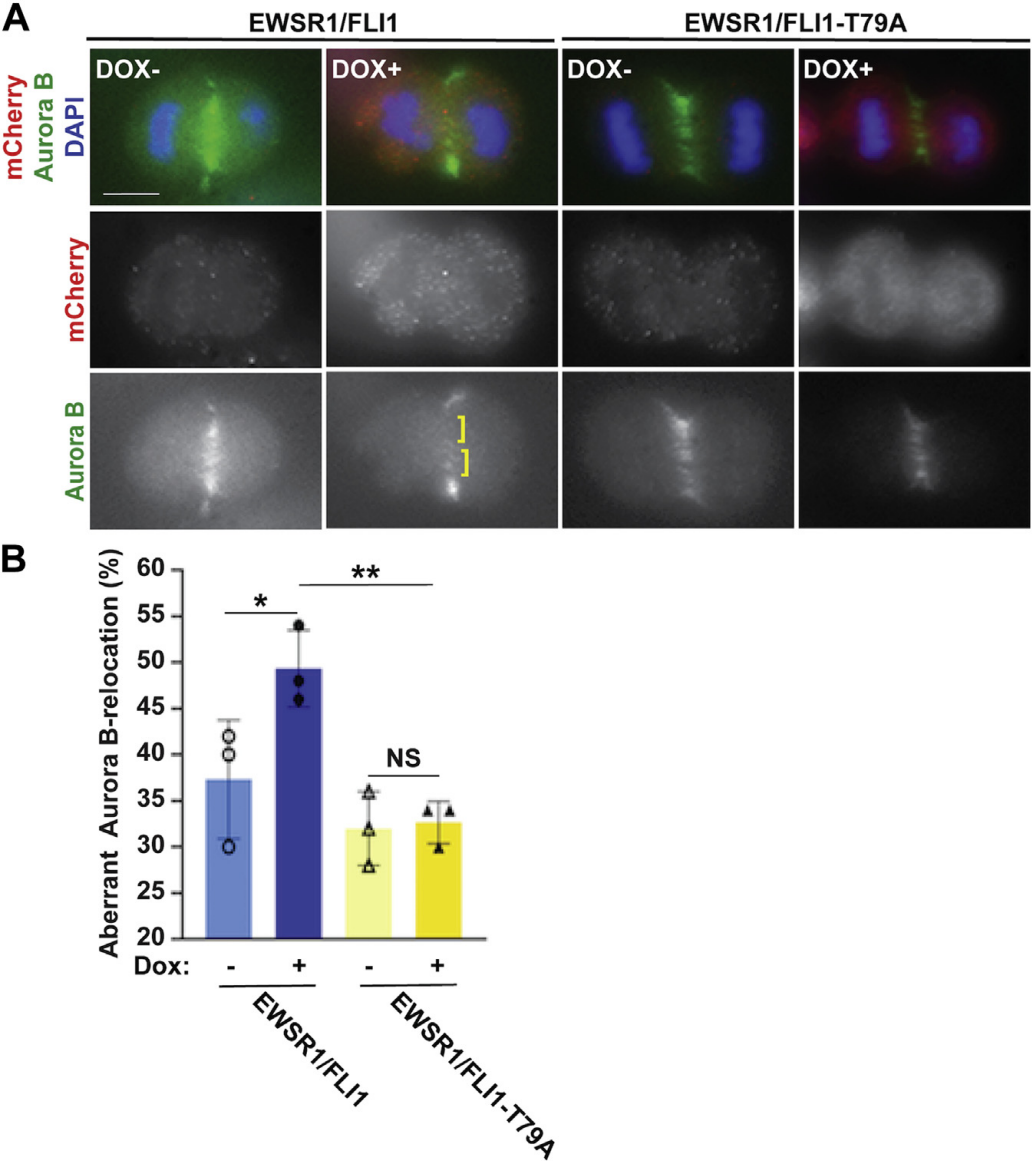
**EWSR1/FLI1-T79 is required for mislocalization of Aurora B at the midzone**. *A*, Representative images of anaphase cells. ]: area with sparse Aurora B signal. Scale bar = 10um. *B*, The percentage of cells that display aberrant Aurora B localization was increased in cells that express *EWSR1/FLI1* but not in cells that express *EWSR1/FLI1-T79A* (50 anaphase cells per sample, n = 3 experiments). ∗*p* < 0.05 and ∗∗*p* < 0.01 (ANOVA one way Tukey HSD).

To determine whether the newly established stable lines recapitulate our previous results of defect on midzone localization of Aurora B using transient expression of EWSR1/FLI1, the stable cell lines were treated with Doxycycline (Dox), followed by immunocytochemistry using an anti-Aurora B antibody and an anti-mCherry antibody to visualize the transgene (30). The localization of Aurora B was analyzed by visually scoring the percentage of cells displaying misaligned Aurora B signal at the midzone. Consistent with our previous report using Ewing sarcoma A673 cells and HeLa cells, the DLD-1 cells that were induced to express EWSR1/FLI1 (Dox+) displayed a greater incidence of aberrant Aurora B localization than uninduced (Dox-) cells (Fig. 4, *A–B*) (25, 30). We scored 50 anaphase cells per sample, in each of three replicate experiments. In Dox-induced cells expressing EWSR1/FLI1, the percentage of cells displaying aberrant Aurora B localization was significantly higher (49 ± 4%) compared with that observed in noninduced (Dox-) cells (37 ± 6%, *p* < 0.05) (Fig. 4*B*). Next, we examined the effect of the T79A residue substitution on midzone formation. The EWSR1/FLI1-T79A expression did not affect the symmetric distribution of Aurora B signal along the midline (Fig. 4*A*). The EWSR1/FLI1-T79A expressing cells did not display a significant change in their incidence of Aurora B mislocalization (33 ± 2%) compared with that observed in noninduced cells (32 ± 4%, *p* > 0.05) (Fig. 4*B*). These results suggest that amino acid Thr 79 of EWSR1/FLI1 is critical to induce mislocalization of Aurora B at the midzone during anaphase.

### EWSR1/FLI1-Thr 79 is required for the induction of aneuploidy

**Figure 5:**
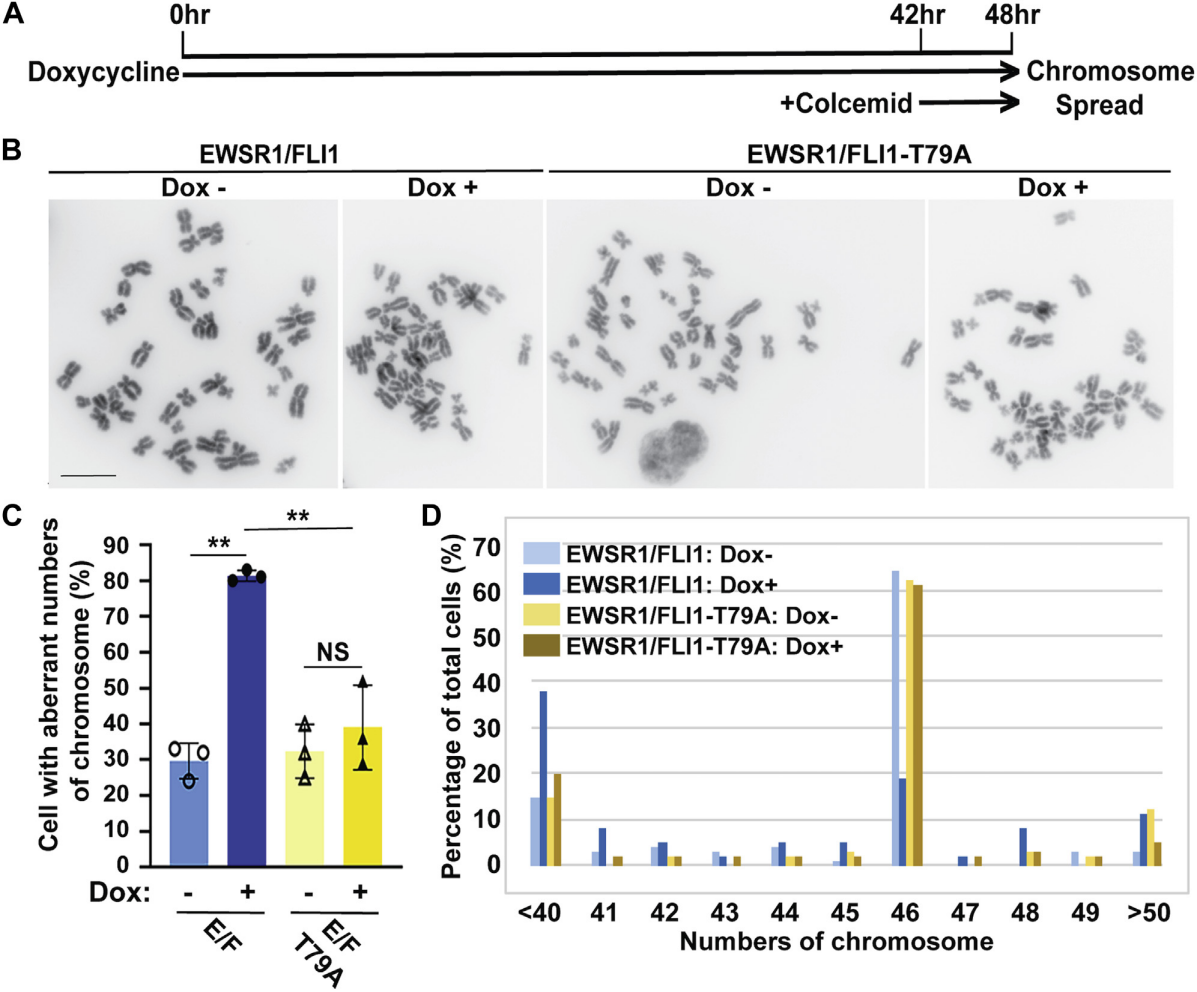
**EWSR1/FLI1-T79 is required for the induction of aneuploidy**. The stable lines were induced with Dox to express either *EWSR1/FLI1* or *EWSR1/FLI1-T79A* (controls left untreated) and subjected to a metaphase chromosome spread protocol. *A*, Schematic for the induction of EWSR1/FLI1 and EWSR1/FLI1-T79A using doxycycline and metaphase synchronization using colcemid. *B*, Representative images of chromosomes from induced cells expressing EWSR1/FLI1 or EWSR1/FLI1-T79A and uninduced cells. (EWSR1/FLI1 Dox-; 46 chromosomes, EWSR1/FLI1 Dox+; 44 chromosomes, EWSR1/FLI1-T79A Dox-; 46 chromosomes, EWSR1/FLI1-T79A Dox+; 46 chromosomes). The color of the chromosome images was inverted using the “Invert” function of Photoshop software, and the background intensity was adjusted using the “Brightness” and “Linear-Curves” functions of Photoshop. Scale bar = 10um. *C*, The percentage of cells that displayed aberrant numbers of chromosomes was increased in cells expressing *EWSR1/FLI1*. (20 cells per sample, n = 3 experiments). ∗∗*p* < 0.01, NS: not significant (ANOVA one way with post-hoc Tukey HSD). *D*, The percentage of cells per sample group containing each chromosome number over the full range of chromosome numbers observed in all cells. Cells expressing *EWSR1/FLI1* have the greatest percentage of nondiploid chromosome numbers.

We investigated whether EWSR1/FLI1 induces aneuploidy and whether Thr 79 is involved in this induction. Because the doubling time of DLD-1 cell is approximately 20 h, and the maximum induction of EWSR1/FLI1 by doxycycline takes approximately 20 h, the stable DLD-1 cell lines were treated with Dox or left untreated for 48 h to assess whether the EWSR1/FLI1 induces aneuploidy after one cell cycle (Fig. 5*A*) (35). The samples were subjected to a metaphase chromosome spreading protocol, followed by visualization of the chromosomes with DAPI. The total number of chromosomes in the cells of each sample group were then scored (Fig. 5*B*). The majority (81 ± 2%) of EWSR1/FLI1 expressing (Dox+) cells contained aberrant numbers of chromosomes, unlike the negative control (Dox-) cells, in which only 30 ± 5% displayed abnormal numbers of chromosomes. Conversely, there was no significant difference in the percentage of cells that displayed aberrant numbers of chromosomes between the EWSR1/FLI1-T79A expressing (Dox+) cells (39 ± 12%) and the control (Dox-) cells (32 ± 8%, *p* > 0.05) (Fig. 5*C*). The majority of cells in the EWSR1/FLI1 (Dox-) sample, the EWSR1/FLI1-T79A (Dox-) sample, and the EWSR1/FLI1-T79A (Dox+) sample had 46 chromosomes (Fig. 5*D*). In contrast, the majority of cells expressing EWSR1/FLI1 (Dox+) had nondiploid chromosome counts (Fig. 5*D*). These results suggest that EWSR1/FLI1 induces aneuploidy and that the Thr 79 residue of EWSR1/FLI1 plays a critical role in this defect.

### Phosphorylation at Thr 79 of EWSR1/FLI1 promotes its localization to the chromosomes

The characterization of the EWSR1/FLI1-T79A cell line suggested that residue Thr 79 of EWSR1/FLI1 plays a critical role in the regulation of Aurora B during metaphase and anaphase and in the induction of aneuploidy. Because Thr is known to be phosphorylated, we sought to determine whether the phosphorylation of amino acid Thr 79 of EWSR1/FLI1 could promote the mislocalization of Aurora B during mitosis and the induction of aneuploidy. To address this, we analyzed the phosphomimetic EWSR1/FLI1-T79D mutant line, which has a Thr to Asp substitution at residue 79, and compared its activity with that of EWSR1/FLI1.

**Figure 6:**
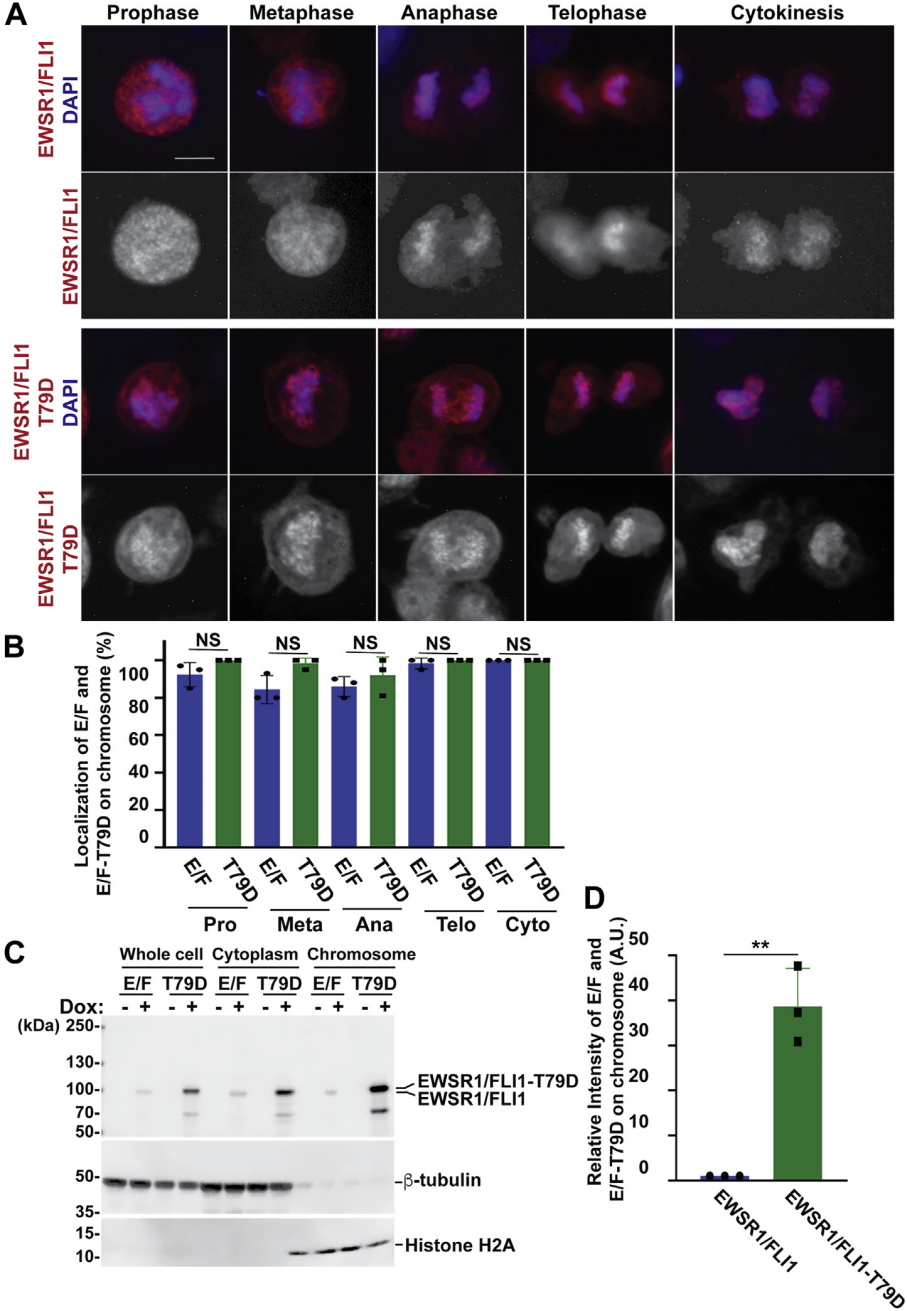
**EWSR1/FLI1-T79D localizes on the chromosomes during early mitosis**. The stable lines were induced with Dox to express either *EWSR1/FLI1* or *EWSR1/FLI1-T79D*, and the cells were mitotically synchronized by thymidine (*A* and *B*, Fig. S1*A*) or by thymidine/nocodazole (*C* and *D*, Fig. S1*B*). *A*, Immunocytochemistry was performed using anti-mCherry primary and Alexa Fluor 596 conjugated secondary antibodies (*red*), and DNA was stained with DAPI (*blue*). The backgrounds of single Z-section images of cells were adjusted using the “Brightness” and “Linear-Curves” functions of Photoshop. The merged images (*top*) and mCherry signal (*bottom*) display the localization of EWSR1/FLI1 and EWSR1/FLI1-T79D on the chromosomes. Scale bar = 10um. *B*, The percentages of cells that displayed localization of either EWSR1/FLI1 or EWSR1/FLI1-T79D on the chromosomes at different phases of mitosis. E/F: EWSR1/FLI1, T79D: EWSR1/FLI1-T79D, Pro: Prophase, Meta: Metaphase, Ana: Anaphase, Telo: Telophase, Cyto: Cytokinesis. n: Total number of cells that were scored. *C*, The lysates were extracted using the same protocol shown in Figure 2*C*, and it were subjected to western blot using anti-FLI1 (*top panel*), anti-β-tubulin (*middle panel*), and anti-H2A antibodies (*bottom panel*). The images were cropped, and color was inverted using the “Invert” function of Photoshop software, and the background was adjusted using the “Brightness” and “Linear-Curves” functions of Photoshop. *D*, Relative intensity of the EWSR1/FLI1 and EWSR1/FLI1-T79D bands (normalized to Histone H2A) from the western blot of the chromosome sample. (n = 3 experiments). ∗∗*p* < 0.01 (Student’s *t*-test).

The localization of EWSR1/FLI1 and EWSR1/FLI1-T79D was analyzed during mitosis. The cells were synchronized using thymidine protocol and released for 9 h (Fig. S1*A*), followed by immunocytochemistry using anti-mCherry antibody, followed by photodocumenting the cells with single Z-section images at each mitotic stage. Similar to EWSR1/FLI1, majority of the EWSR1/FLI1-T79D-mCherry expressing cells displayed strong mCherry signals on the chromosomes during all mitotic stages (Fig. 6, *A–B*). We further analyzed the localization of EWSR1/FLI1-T79D in biochemically fractionated cytoplasm and chromosome samples using the same protocol used in the experiments shown in Figure 2, *C* and *D*. Consistent with the result obtained by immunocytochemistry, both EWSR1/FLI1 and EWSR1/FLI1-T79D signal are present in the chromosome fraction in Dox-treated cells (Fig. 6*C*). Interestingly, quantification of the band intensity for the EWSR1/FLI1 and EWSR1/FLI1-T79D signals in the chromosome fractions revealed that the signal intensity for EWSR1/FLI1-T79D was significantly higher than that of EWSR1/FLI1 (defined as 1.0) (Fig. 6*D*). Despite the higher expression level of the EWSR1/FLI1-T79A and EWSR1/FLI1-T79D than EWSR1/FLI1 (Figs. 2*C* and 6*C* whole cell lysate), the localization of EWSR1/FLI1-T79A on the chromosome was significantly lower than that of EWSR1/FLI1. Together, these results suggest that the phosphorylation of the residue Thr 79 of EWSR1/FLI1 promotes its localization to chromosomes during mitosis.

**Figure 7:**
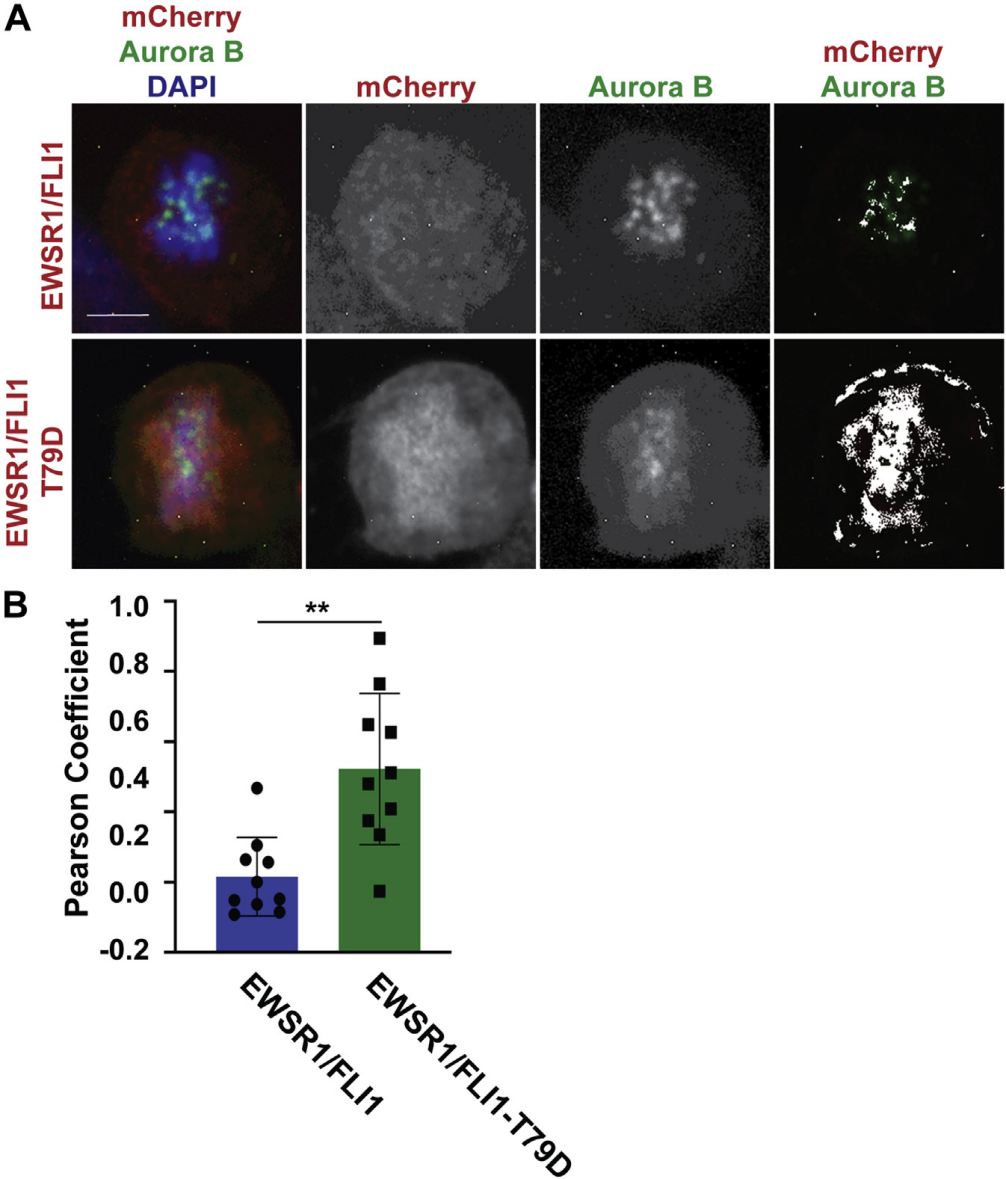
**EWSR1/FLI1-T79D display colocalization with Aurora B during metaphase**. *EWSR1/FLI1* and *EWSR1/FLI1-T79D* expressing cells (Dox+) and control (Dox-) cells were synchronized in mitosis using thymidine (Fig. S1*A*), and subjected to immunocytochemistry using anti-mCherry primary and Alexa Fluor 594 (*red*), and anti-Aurora B primary and Alexa Fluor 488 conjugated secondary antibodies (*green*). *A*, Merged single Z-section images from mCherry/Aurora B/DAPI (*left*), with mCherry (*middle*), with Aurora B (*middle*), and merged images from mCherry/Aurora B (*white* represents the areas with colocalized Aurora B (*green*) and mCherry (*red*) signals) (*right panel*). Scale bar = 10um. *B*, The Pearson Coefficient of covariance for Aurora B (*green*) and mCherry (*red*) signals in Dox-treated metaphase cells. There is significant difference between cells expressing EWSR1/FLI1 and cells expressing EWSR1/FLI1-T79D. ∗∗*p* < 0.01 (Student’s *t*-test).

We further examined whether EWSR1/FLI1-T79D and Aurora B colocalize on metaphase chromosomes. Using the same protocol described in Fig. S1*B*, the stable cell lines were treated with Dox and thymidine/nocodazole. The EWSR1/FLI1 and EWSR1/FLI1-T79D expressing DLD-1 cell lines were subjected to immunocytochemistry using anti-mCherry and Alexa Fluor 594 antibodies and anti-Aurora B and Alexa Fluor 488 antibodies, followed by the same colocalization analysis as Figure 3 using the Pixelmap function of Fiji Imaging software. In contrast to the results shown in Figure 3 with T79A mutant, there was a significant increase in the Pearson Coefficient between EWSR1/FLI1 (n = 10 cells) and EWSR1/FLI1-T79D (n = 10 cells) (*p* < 0.01) (Fig. 7, *A–B*). There was higher level of colocalization between EWSR1/FLI1-T79D and Aurora B compared with that of EWSR1/FLI1 and Aurora B. The result suggests that the phosphorylation of residue Thr 79 of EWSR1/FLI1 is essential for the colocalization of EWSR1/FLI1 and Aurora B.

### Phosphorylation of EWSR1/FLI1-Thr 79 is critical for induction of the aberrant localization of Aurora B at the midzone during anaphase

**Figure 8:**
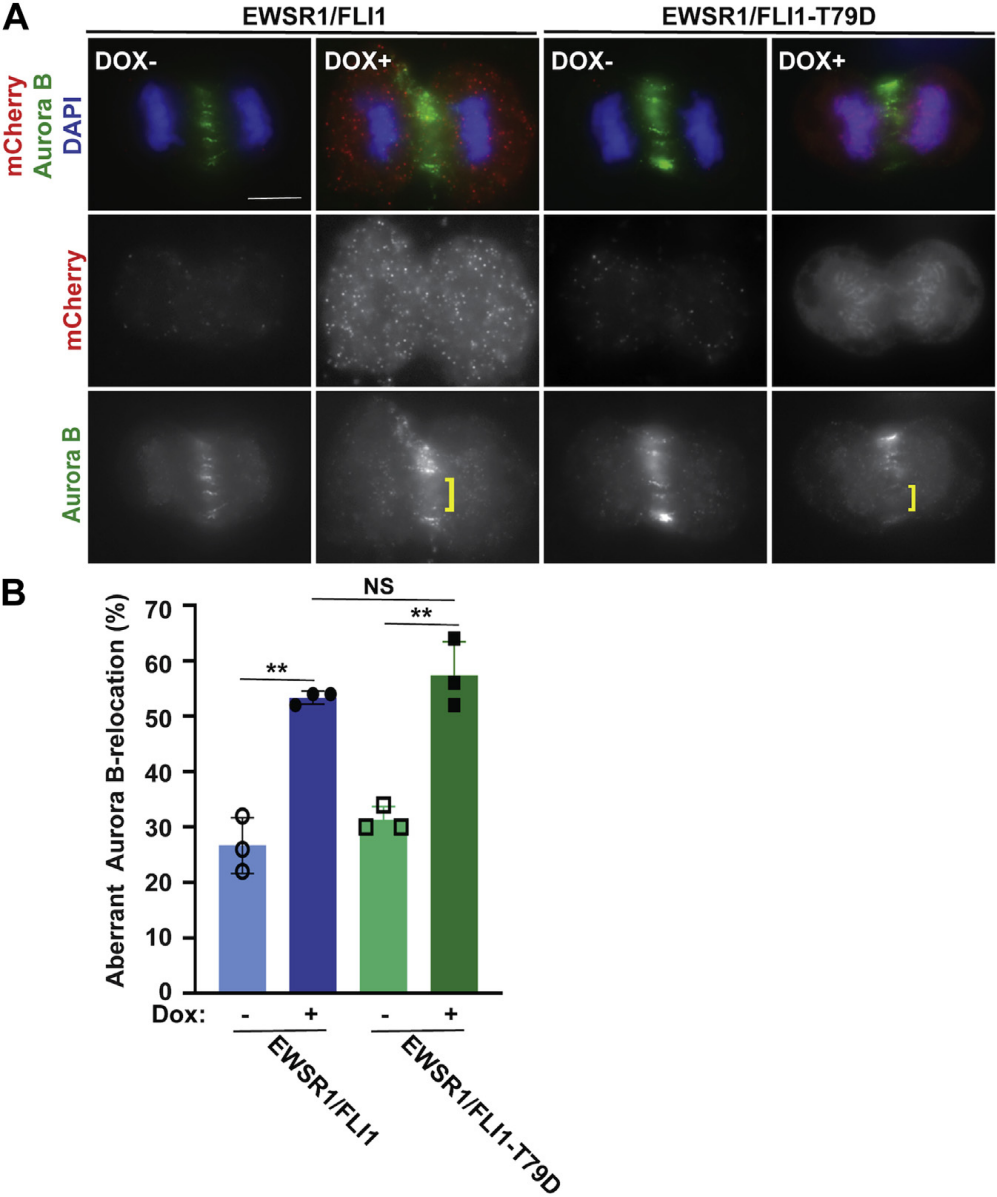
**EWSR1/FLI1-T79D promotes mislocalization of Aurora B at the midzone**. *A*, Representative images of anaphase cells. [: area with unevenly distributed Aurora B signal. Scale bar = 10um. *B*, The percentage of cells that display aberrant Aurora B localization was higher in cells that express *EWSR1/FLI1-T79D* (50 anaphase cells per sample, n = 3 experiments) than in control cells. ∗*p* < 0.05 and ∗∗*p* < 0.01 (ANOVA one way with post-hoc Tukey HSD).

Because EWSR1/FLI1-T79A lacks the ability to localize Aurora B to the midzone, we investigated the effect of phosphorylation of EWSR1/FLI1 residue T79 on Aurora B localization using stable cell lines with the phosphomimetic mutant EWSR1/FLI1-T79D. DLD1 cells expressing EWSR1/FLI1 (Dox+) and EWSR1/FLI1-T79D (Dox+) (and untreated controls) were subjected to immunocytochemistry using anti-mCherry and anti-Aurora B antibodies. Cells expressing either EWSR1/FLI1 or EWSR1/FLI1-T79D displayed a greater incidence of aberrant Aurora B localization than uninduced (Dox-) cells (Fig. 8*A*) (25, 30). We visually scored the localization of Aurora B at the midzone in 50 anaphase cells per sample (n = 3 experiments). As was shown in Figure 4, the percentage of cells that displayed aberrant Aurora B localization was significantly higher (53.3 ± 1.2 %) in cells that express EWSR1/FLI1 than that observed in noninduced (Dox-) cells (26.7 ± 5.0 %, *p* < 0.01). Cells expressing the phosphomimetic EWSR1/FLI1-T79D also displayed a significant increase in their incidence of Aurora B mislocalization (57.3 ± 6.1 %) compared with that observed in noninduced cells (31.3 ± 2.3 %, *p* < 0.01) (Fig. 8*B*). Moreover, there was no significant difference between the incidence of aberrant Aurora B localization in EWSR1/FLI1-expressing cells and EWSR1/FLI1-T79D-expressing cells (*p* > 0.05, nonsignificant). These results suggest that phosphorylation of the Thr 79 residue of EWSR1/FLI1 is critical for the EWSR1/FLI1-induced mislocalization of Aurora B at the midzone during anaphase.

### Phosphorylation of EWSR1/FLI1-Thr 79 is critical to the induction of aneuploidy by EWSR1/FLI1 expression

**Figure 9:**
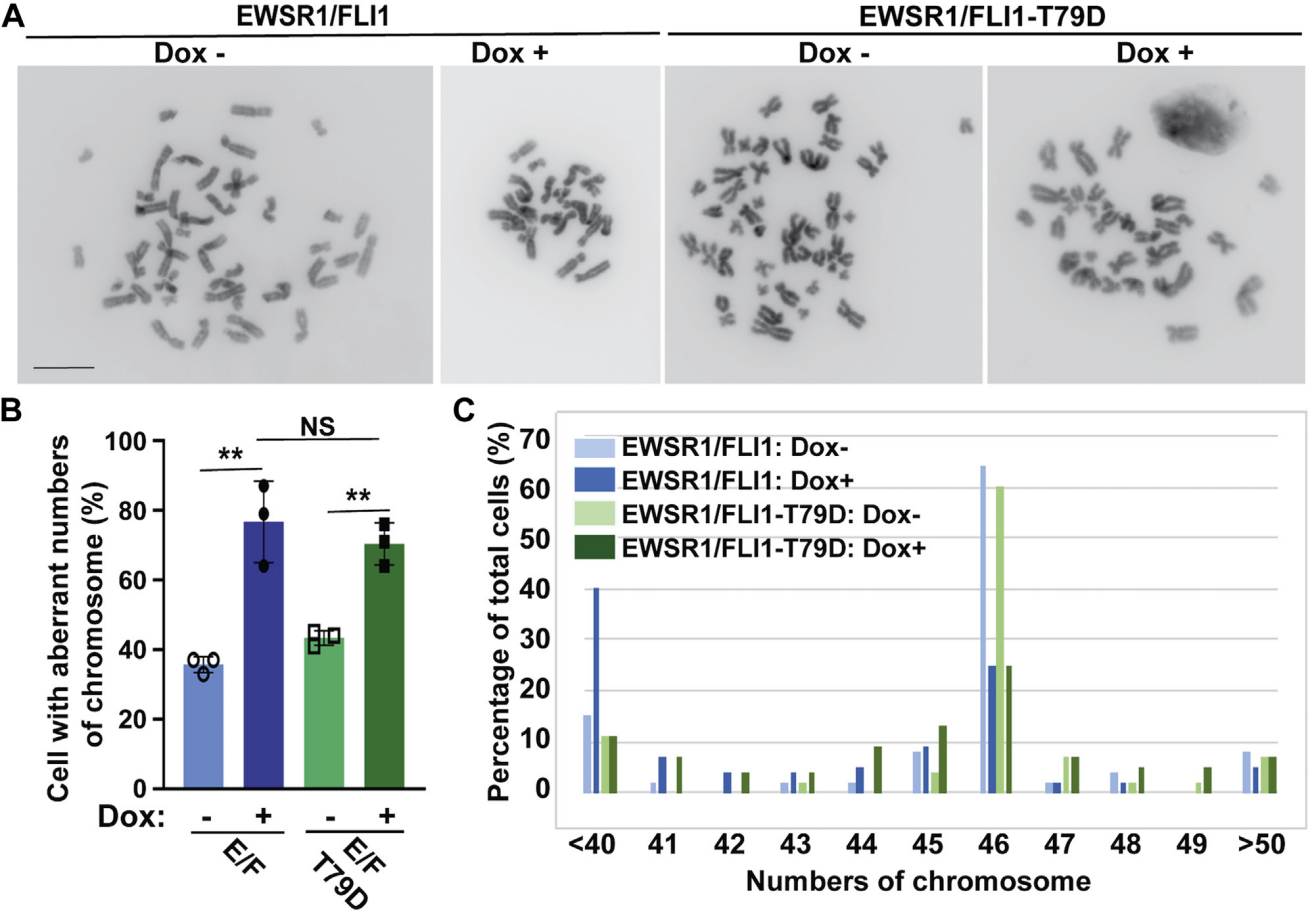
**EWSR1/FLI1-T79D promotes the induction of aneuploidy**. *EWSR1/FLI1* and *EWSR1/FLI1-T79D* expressing cells (Dox+) and controls (Dox untreated) were subjected to a metaphase chromosome spread protocol (Fig. 5*A*). *A*, Representative images of chromosomes from induced cells expressing EWSR1/FLI1 or EWSR1/FLI1-T79D and uninduced cells. (EWSR1/FLI1 Dox-; 46 chromosomes, EWSR1/FLI1 Dox+; 23 chromosomes, EWSR1/FLI1-T79D Dox-; 46 chromosomes, EWSR1/FLI1-T79D Dox+; 30 chromosomes). The color of the chromosome images was inverted using the “Invert” function of Photoshop software. The background intensity was adjusted using the “Brightness” and “Linear-Curves” functions of Photoshop. Scale bar = 10um. *B*, The percentage of cells that displayed aberrant numbers of chromosomes was increased in cells expressing *EWSR1/FLI1-T79D,* with no significant difference in the incidence of nondiploid chromosome numbers between cells expressing *EWSR1/FLI1* and cells expressing *EWSR1/FLI1-T79D*. (20 cells per sample, n = 3 experiments). ∗∗*p* < 0.01, NS: not significant (ANOVA one way with post-hoc Tukey HSD). *C*, The percentage of cells per sample group containing each chromosome number over the full range of chromosome numbers observed in all cells.

To address whether phosphorylation of EWSR1/FLI1 at Thr 79 is a critical modification required for the induction of aneuploidy in cells expressing EWSR1/FLI1, chromosome spread experiments were performed on Dox-treated cells expressing EWSR1/FLI1 and EWSR1/FLI1-T79D (Fig. 9*A*). Consistent with the results shown in Figure 5, cells expressing EWSR1/FLI1 had a higher incidence of aberrant numbers of chromosomes (76.7 ± 11.7 %) than Dox negative cells (35.7 ± 2.3 %, *p* < 0.01) that don’t express the fusion protein. Surprisingly, Dox+ cells expressing EWSR1/FLI1-T79D also displayed a higher incidence of aberrant numbers of chromosomes (70.3 ± 6.0 %) compared with Dox negative cells (43.3 ± 2.1 %, *p* < 0.01), and there was no significant difference in the incidence of cells containing aberrant chromosome numbers between cells expressing EWSR1/FLI1 and cells expressing the phosphomimetic mutant EWSR1/FLI1-T79D (Fig. 9*B*). The number of chromosomes per cell in cells expressing EWSR1/FLI1 and cells expressing EWSR1/FLI1-T79D are shown in Figure 9*C*. These results suggest that EWSR1/FLI1 induces aneuploidy when the Thr 79 residue of EWSR1/FLI1 is phosphorylated.

**Figure 10:**
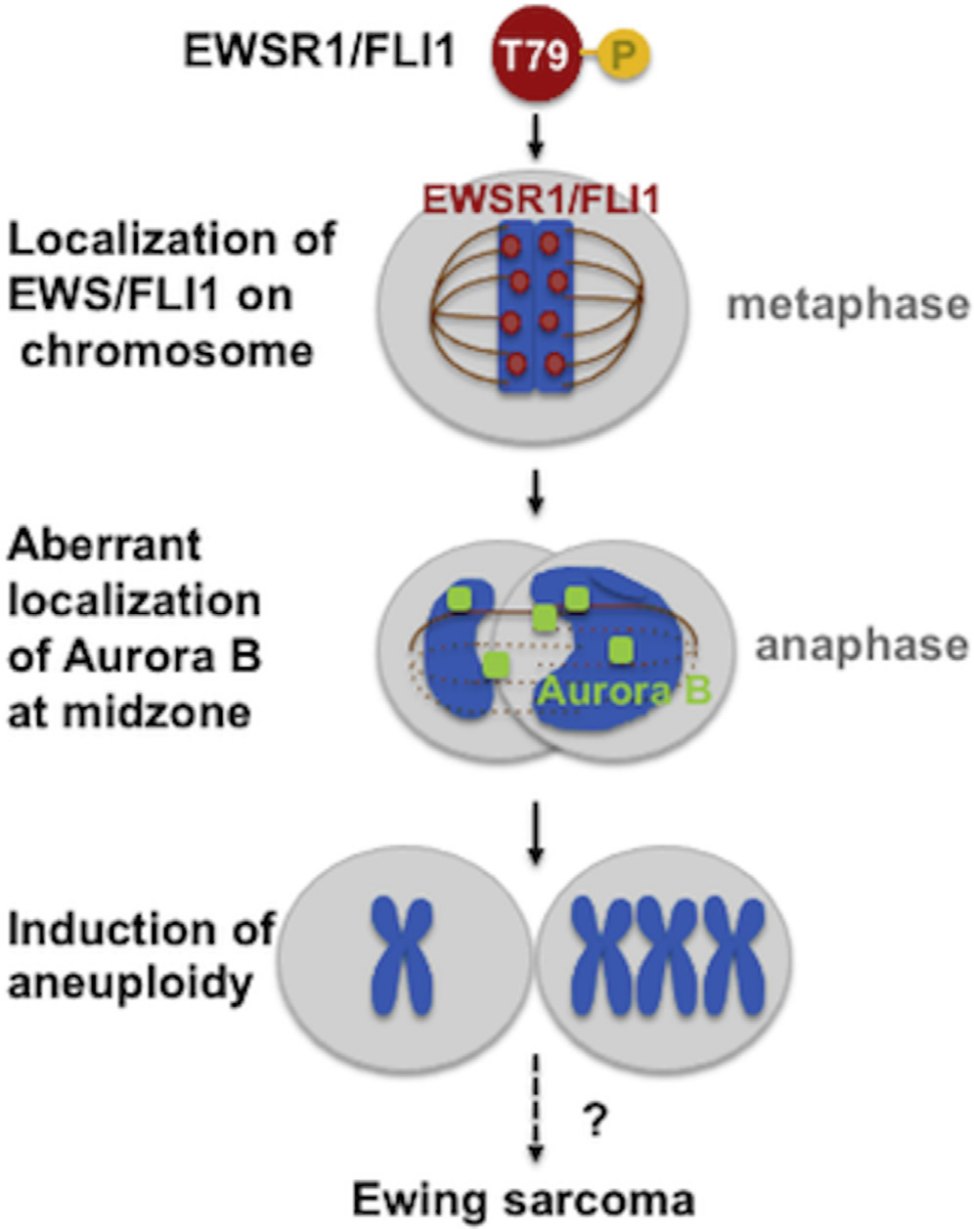
**Schematic model of the study**. The phosphorylation of EWSR1/FLI1 leads to the localization of EWSR1/FLI1 on mitotic chromosomes during early mitosis, with impaired localization of Aurora B at the midzone and induction of aneuploidy after a single cell cycle. The result suggests that the phosphorylation of amino acid Thr 79 of EWSR1/FLI1 has critical activity in mediating this pathway.

Together, these studies demonstrate that phosphorylation of Thr 79 residues of EWSR1/FLI1 promotes its localization to mitotic chromosomes during prophase and metaphase, with impaired localization of Aurora B at the midzone and induction of aneuploidy (Fig. 10). The conversion of a single amino acid at residue 79 of EWSR1/FLI1 from Thr to Ala was sufficient to abolish the induction of the phenotypes described above. In contrast, the substitution of amino acid 79 of EWSR1/FLI1 from Thr to Asp did not abolish the induction of the phenotypes described above. Furthermore, our results suggest these phenotypes are mediated through a single pathway: the localization of EWSR1/FLI1 to the chromosomes during prophase and metaphase triggers defects in the midzone and ultimately leads to the induction of aneuploidy (Fig. 10).

## Discussion

Our results show the first direct evidence for an association between the localization of EWSR1/FLI1 on mitotic chromosomes during early mitosis, impaired localization of Aurora B at the midzone, and induction of aneuploidy. Furthermore, we demonstrate that the phosphorylation of Thr 79 of EWSR1/FLI1 is critical to mediate this pathway.

Investigation of the upstream regulatory mechanisms that control the phosphorylation of EWSR1/FLI1-Thr 79, thereby regulating its activity and promoting mitotic dysfunction, is essential for understanding the pathogenesis of Ewing sarcoma. A previous study demonstrated that DNA damage promotes the p38α/p38β MAPK-dependent phosphorylation of Thr 79 of EWSR1/FLI1 (31). Therefore, to understand the mechanisms involved in pathogenesis, it is essential to determine whether the same kinase mediates phosphorylation during mitosis. In addition, phosphorylation and O-GlcNAcylation often function as “molecular switches” because Thr can undergo both modifications. This is a plausible mechanism for regulating the function of the fusion protein. Our previous report demonstrated that knockdown of OGA (the enzyme that removes O-GlcNAc from proteins) in HeLa cells resulted in increased levels of O-GlcNAcylation of EWSR1 as well as aberrant localization of EWSR1 during metaphase (36). Because both EWSR1 and EWSR1/FLI1 undergo O-GlcNAcylation, and because EWSR1/FLI1 share the same sequence as the N-terminus of EWSR1, (including Thr 79), it is possible that EWSR1/FLI1 activity is controlled by O-GlcNAcylation at Thr 79 (32, 37). With our demonstration of the importance of T79 in the induction of aneuploidy, the EWSR1/FLI1-T79A and EWSR1/FLI1-T79D mutants that we developed may provide an important tool for dissecting the disease. Interestingly, numerous proteins are known to alternatively undergo both O-GlcNAcylation and phosphorylation at the same Thr residue, and the phenotypes of the cells expressing those modified proteins are altered by the posttranslational modification (PTM). Therefore, it is possible that EWSR1-T79 and EWSR1/FLI1-T79 undergo both phosphorylation and O-GlcNAcylation and that the phosphorylation of EWSR1/FLI1 is the dominant PTM critical for the induction of mitotic dysfunction. Whether phosphorylation or O-GlcNAcylation of EWSR1/FLI1 is involved in the induction of aneuploidy and how these PTMs may alter protein function is yet to be determined.

Elucidating whether endogenous EWSR1 plays a role in this process is important because several studies have shown that EWSR1/FLI1 interacts with and inhibits the function of EWSR1 (30, 38, 39). Our previous studies demonstrated that EWSR1 has a role in mitosis, promoting the proper localization of Aurora B to the midzone through biochemical interaction between the two molecules (30). In addition, EWSR1 functions in maintaining chromosome stability and preventing tumorigenesis in zebrafish (40). To understand the mechanism of impaired Aurora B localization, it is necessary to determine the interactions between EWSR1, Aurora B, and EWSR1/FLI1. It is possible that EWSR1/FLI1, EWSR1 and Aurora B form a complex or that dimers formed between EWSR1/FLI1 and Aurora B compete with dimers of EWSR1-Aurora B. In addition, it is also essential to understand whether these biochemical interactions take place on the chromosome. Given the critical importance of amino acid T79 in the function of EWSR1/FLI1, utilizing the amino acid residue-substituted EWSR1/FLI1-T79A and EWSR1/FLI1-T79D constructs will further improve our understanding of the molecular pathogenesis of the disease. For example, because EWSR1/FLI1-T79A does not localize to chromosomes during early mitosis, we can determine if this molecule lacks the ability to interact with EWSR1 and/or Aurora B. Alternatively, EWSR1/FLI1-T79A may form dimers with EWSR1 or Aurora B, but may not be capable of localizing to chromosomes, indicating that localization is mediated by an additional unknown mechanism. Conversely, it is possible that the EWSR1/FLI1-T79D has an opposite activity compared with EWSR1/FLI1-T79A. Furthermore, while our data points to the role of EWSR1/FLI1 in mitosis, it is necessary to address whether the well-described functions of EWSR1/FLI1 in transcription and splicing affect the induction of aneuploidy through impairment of Aurora B in mesenchymal stem cells, the cell of origin for Ewing sarcoma, or in an animal model (41, 42).

Future studies will be required to elucidate the mechanism for the enrichment of aneuploidy of specific chromosomes (*e.g.*, the high incidence (∼50%) of trisomy of chromosome 8) observed in Ewing sarcoma cells. Our results suggested that phosphorylation of Thr 79 of EWSR1/FLI1 promotes the induction of aneuploidy, and the daughter cells of cells expressing EWSR1/FLI1 or EWSR1/FLI1-T79D displayed a wide range of chromosome numbers (Fig. 9). One possible explanation is that there is no selectivity for specific chromosomes in the EWSR1/FLI1-dependent induction of aneuploidy, but during subsequent cell divisions, cells that inherit particular chromosomes manage to survive and expand during Ewing sarcoma development. Now that we have demonstrated the importance of phosphorylation of EWSR1/FLI1-T79 in the induction of aneuploidy, future studies will investigate the mechanism leading to disease-specific changes in chromosome numbers. It will be essential to utilize human mesenchymal stem cells (hMSC), the cells of origin for Ewing sarcoma to study the enrichment of specific chromosome gains and losses (41, 43). There are two advantages of utilizing hMSC in the study. First, hMSC will display high penetrance of phenotypes due to its normal genetic background. For this reason, it is possible to study the incidence of aneuploidy of specific chromosome. Second, clonal selection of the cell will be influenced by the cell type. Because the hMSC is a cell of origin of Ewing sarcoma, it allows to study the process of clonal selection. For these reasons, the future study using hMSC allows to model the induction of aneuploidy in a specific chromosome and the process of its clonal selection during Ewing sarcoma development.

This study provides a platform for the future study of how EWSR1 fusion proteins impair Aurora B function and induce aneuploidy. Utilizing the EWSR1/FLI1-T79A and EWSR1/FLI1-T79D mutant constructs as well as constructs involving EWSR1-T79A and EWSR1-T79D fused to other genes will enable us to understand the role of phosphorylation in various disease phenotypes. Because various types of sarcomas express fusion genes that have N terminus of EWSR1 including Thr 79, this study has the potential to reveal a universal mechanism for the pathogenesis underlying all EWSR1 fusion protein-expressing sarcomas. Identification of the kinase that modulates the activity of Thr 79 of EWSR1/FLI1 fusion protein and associated with chromosome instability (CIN) may lead to the discovery of new therapeutic approaches.

## Experimental procedures

### Donor plasmid construction

Human *pSG5-2xFLAG-EWSR1/FLI1-T79A* and *pSG5-2xFLAG-EWSR1/FLI1-T79D* was constructed from the plasmid *pSG5-2xFLAG-EWSR1/FLI1* following the manufacturer’s protocol (QuickChange II Site-Directed Mutagenesis Kit, #200524, Agilent Technologies), using the primers:

- *EWSR1/FLI1-T79A:F* (5′- CCCACTGGTTATACTGCTCCAACTGCCCCCC -3′)
- *EWSR1/FLI1-T79A:R* (5′- GGGGGGCAGTTGGAGCAGTATAACCAGTGGG -3′).
- *hEWS/FLI1-T79D:F*: 5'-CCCACTGGTTATACTgatCCAACTGCCCCC-3'
- *hEWS/FLI1-T79D:R*:5'-GGGGGCAGTTGGatcAGTATAACCAGTGGG-3'

Then, the h*EWSR1/FLI1,* h*EWSR1/FLI1-T79A*, and h*EWSR1/FLI1-T79D* genes are amplified by PCR from *pSG5-2xFLAG-EWSR1/FLI1, pSG5-2xFLAG-EWSR1/FLI1-T79A*, and h*EWSR1/FLI1-T79D* respectively, using the following primers that contain a linker with MluI and NotI sites.

- *MluI hEWS:F* (5’-GATACGCGTATGGCTGCCACGGATTAC-3’)
- *NotI nostp hsEF:R* (5’-CTGCGGCCGCGTAGTAGCTGCCTAAGTG-3’)

Concurrently, the mCherry gene was amplified from the pCDNA4-His-maxC-mCherry vector using the following primers that contain a linker with NotI and SalI (44).

- *NotI mChe: F* (5’-GCGGCCGCAGGCGCTGG-3')
- *SalI stop mChe:R* (5’-CTTGTCGACTTACTTGTACAGCTCGTCC-3’)

A new multicloning site was generated in the plasmid pMK243 (Tet-OsTIR1-PURO) (Addgene) by excising the OsTIR1 fragment with BglII and MluI, then inserting an oligo containing BglII-SalI-AgeI-EcoRV-MluI sites. Finally, the DNA transgenes of *EWSR1/FLI1, EWSR1/FLI1-T79A*, or *EWSR1/FLI1-T79D* and *mCherry* were ligated into the MluI and SalI sites of the newly modified pMK243 plasmid (45). The mutations were introduced in PAM sequences on the homology arms (46).

### Establishment of stable cell lines

The targeted integration of exogenous sequences of donor plasmids was accomplished using the CRISPR/Cas9 system (29). DLD-1 cells were plated in 3.5 cm dishes and transfected with the donor and guide RNA plasmids targeting the AAVS1 locus (from Addgene, AAVS1 T2 CRISPR in pX330, #72833) using ViaFect (Promega, #E4981) following the manufacturer’s protocol (29, 33). The cells were cultured for 2 days, then plated in 10 cm dishes at less than 20% confluency in 1ug/ml puromycin containing selection media. The cells were cultured for 10–14 days with selection media, then colonies were isolated and grown in 48-well plates. When the cells became confluent, they were harvested, and the expression of the transgene was verified by western blot and immunocytochemistry. Sixteen clones were isolated from *EWSR1/FLI1* transfected cells, and 11 clones expressed the transgene. Twenty-four clones were isolated from *EWSR1/FLI1-T79A* transfected cells, and five clones expressed the transgene. In addition, 24 clones were isolated from *EWSR1/FLI1-T79D* transfected cells, and six clones expressed the transgene.

### Cell culture and induction of *EWSR1/FLI1*, *EWSR1/FLI1-T79A* and *EWSR1/FLI1-T79D* expression using doxycycline

DLD-1 cell lines were maintained in McCoy’s 5A Medium with 10% (vol/vol) Tet System Approved FBS, at 37 °C with 5% (vol/vol) CO_2_. The expression of *EWSR1/FLI1*, *EWSR1/FLI1-T79A*, and *EWSR1/FLI1-T79D* was induced in the DLD-1 cells by treatment with 1ug/ml Doxycycline (Dox) for 20 h. All cells used in this study were passaged less than 20 times.

### Cell synchronization

The cells were synchronized in mitosis using 2 mM Thymidine for 18 h, followed by washing three times with McCoy's 5A 1X L-glutamine medium (without FBS). The medium was replaced with 10% FBS containing medium and the cells were incubated for 9 h. The doxycycline was added to the cell culture medium for 20 h prior to the harvesting the cell (Fig. S1*A*).

The cells were synchronized in mitosis using an established Thymidine/Nocodazole cell cycle arrest protocol with minor modifications (47). Specifically, the cells were arrested with 2 mM Thymidine for 18 h, then released from the Thymidine block by washing three times with McCoy's 5A 1X L-glutamine medium (without FBS). Then the medium was replaced with 10% FBS containing medium and the cells were incubated for 6 h. Next, the cells were treated with 100 ng/ml of Nocodazole for 6 h, and the mitotic cells were collected by performing a mitotic shake-off, followed by three washes with McCoy's 5A 1X L-glutamine medium. The mitotic cells were rereleased for 30 min by plating in culture dishes or on coverslips, and the cells were subjected to the described experiments. Concurrently, the cell were treated by doxycycline for 20 h prior to the fixation (Fig. S1*B*).

### Immunocytochemistry

The mitotically synchronized cells were plated onto fibronectin coated coverslips (NEUVITRO, #GG-12–1.5-Fibronectin), released for 30 min, and subjected to immunocytochemistry as described previously, with minor modifications (25). The following antibodies were used: Rabbit anti-mCherry (1:500 dilution) (Abcam, #ab167453); Mouse anti-Aurora B (AIM1) (1:250 dilution) (BD Biosciences, #611082); anti-Mouse Alexa Fluor 488 (1:500 dilution) (Invitrogen, #M11032); and anti-Rabbit Alexa Fluor 594 (1:500 dilution) (Invitrogen, #M32731) (25). All cells were counterstained with DAPI/VECTASHIELD mounting media (VECTOR laboratories, #H-1200). Cell images were documented at 1000× magnification with a Nikon Ti Eclipse microscope using MetaMorph imaging software. The Z-section images of Figure 2 and Figure 3 were also documented at 1000× magnification using OptiGrid Structured Illumination microscopy (Nikon). The Z-section images of Figure 6 and Figure 7 were documented at 1000× magnification using Yokogawa CSU10 Spinning Disc Structured Illumination microscopy (Olympus).

### Cell fractionation

Cells were synchronized in mitosis, then harvested, and lysed as described previously (48). Each sample lysate was subjected to centrifugation (500 X g for 3 min at 4 °C), and the supernatant was used as the cytoplasm fraction. The pellet was resuspended in lysis buffer, layered over a 40% glycerol solution, and subjected to centrifugation (10,000 X g for 5 min at 4 °C). The pellet from this step was subjected to the same process once more, and the pellet from this final centrifugation was used as the “chromosome” fraction.

### Western blotting

Cell lysates from both Dox-treated and untreated cells were subjected to western blotting using a 1:1000 dilution of rabbit anti-human FLI1 (Thermo Scientific, #PA1-21023), or 1:2500 dilution of mouse anti-β-tubulin (Sigma-Aldrich, T4026) as primary antibodies, followed by treatment with a 1:100000 dilution of IRDye 680RD donkey anti-mouse IgG secondary antibody (LI-COR, #926–68072) and IRDye 680RD donkey anti-Rabbit IgG (LI-COR, #926–68073).

The fractionated cell samples obtained from Dox-treated and untreated stable cells were subjected to western blotting using a 1:1000 dilution of Rabbit anti-human FLI1 (Thermo Scientific, #PA1-21023), followed by treatment with a 1:10,000 dilution of IRDye 680RD donkey anti-Rabbit IgG (LI-COR, #926–68073). The western blot of the loading controls was treated with a 1:1000 dilution of rabbit anti-Histone H2A (Abcam, #ab18255) and a 1:2000 dilution of mouse anti-β-tubulin (Sigma-Aldrich, T4026) antibodies. All images of western blots were captured using the LI-COR Odyssey Imaging System.

### Metaphase chromosome spreads

Both Dox-induced and uninduced cells were treated with 40 ng/ml colcemid when the cells were at 40% confluency. The cells were incubated for 6 h, then washed three times with PBS, trypsinized and harvested into centrifuge tubes, and treated with 500ul of 0.025 M NaCitrate at 37 °C for 20 min. Next, 3 ml of ice-chilled methanol:acetic acid (3:1) solution was added to the cell suspension while the tubes were vigorously shaken using a vortex. The cells were then centrifuged at 4000 rpm for 5 min, resuspended in 1 ml of ice-chilled methanol:acetic acid (3:1) solution, and incubated for 10 min on ice; this process was repeated twice more. Finally, the cells were resuspended in 100ul of methanol:acetic acid (3:1) solution, and this cell suspension was dropped onto glass slides. The DNA on the slides was counterstained with DAPI/VECTASHIELD mounting media. The images of chromosomes were documented at 1000× magnification with a Nikon Ti Eclipse microscope using MetaMorph Imaging software.

### Statistics

Standard deviation (SD) is shown as error bars for each graph. The statistical confidence was defined at *p* < 0.05 by ANOVA one-way analysis followed by Tukey HSD (Honestly Significant Difference).

## Data availability statement

All data are indicated in the manuscript.

## Conflict of interest

The authors declare that they have no conflicts of interest with the contents of this article.
