# Supplementary material for: Chromosomal localization of Ewing sarcoma EWSR1/FLI1 protein promotes the induction of aneuploidy
Source: J Biol Chem. 2020 Dec 10;296:100164. doi: 10.1074/jbc.RA120.014328 (PMC7857440; doi:10.1074/jbc.RA120.014328)
Supplement: Figure S1 [file mmc1.pdf]

Fig S1

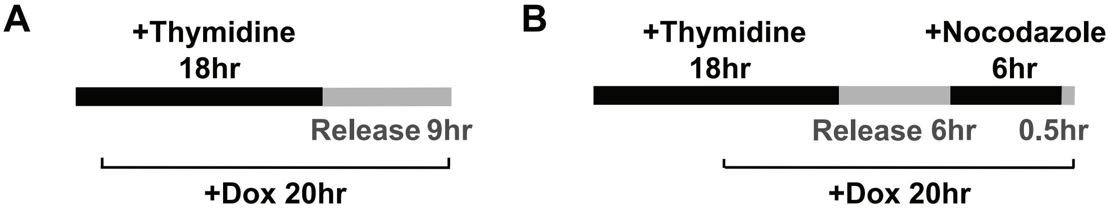

Fig S1. Schematic time line of the mitotic synchronization and the Dox treatment. The stable lines were induced with Dox to express *EWSR1/FLI1*, *EWSR1/FLI1-T79A* and *EWSR1/FLI1-T79D*, and synchronized to mitosis using thymidine (**A**) or thymidine/nocodazole (**B**). **A**. Schematic for mitotic synchronization using thymidine. **B**. Schematic for mitotic synchronization using thymidine and nocodazole.
